# The brain trancytosome: pooled in vivo screening of 1000s of biologics discovers novel “portal” receptors and mechanisms for macromolecule CNS delivery

**DOI:** 10.1002/alz70859_103983

**Published:** 2025-12-25

**Authors:** Kimberly Scearce‐Levie, Pierce Ogden, Isabel Albuja, Fabliha Anam, Anisha Anand, John Avera, Allie Braun, Sean Carroll, Marshall Case, Karen Duffy, Alvaro Garcia, Alex Gerew, Jack Gourdeau, Hala Haddad, Glenn Hauk, Yendee Ho‐Rath, Gleb Kuznetsov, Shane Lofgren, Ainaz Malekoltojari, Roasa Mehmood, Naveen Mehta, Mike Nichols, Maggie Prescott, Supriva Ravichandran, Alex Reis, Manan Shah, Tianxiao Sun, Erik Swanson, Brian Wipke, Kate Nudel

**Affiliations:** ^1^ Manifold Bio, Boston, MA USA

## Abstract

**Background:**

Central nervous system (CNS) diseases are difficult to treat due to the blood‐brain barrier (BBB), which prevents many therapeutics from reaching the brain. Receptor‐mediated transcytosis (RMT) shows promise for transporting macromolecules across the BBB, but current targeting methods lack CNS specificity and cell‐type selectivity. The number of validated "Portals" (BBB receptors with demonstrated RMT *in vivo*) is limited due to historical screening constraints.

**Method:**

We conducted the most comprehensive in vivo evaluation of BBB shuttles and Portals to date, screening over 3,000 candidates against 68 potential Portal targets in mice. Our novel pooled in vivo screening technology, mCodes™, allowed us to simultaneously assess quantitative tissue distribution for up to 100 molecules per animal with high precision.

**Result:**

This approach revealed the "Transcytosome," an expanded set of Portal targets capable of facilitating RMT across the BBB. Detailed pharmacokinetic and biodistribution analyses of select novel Portals demonstrate distinct properties compared to well‐characterized targets like transferrin receptor (TfR) and CD98. Novel portals were discovered that demonstrated optimal characteristics for therapeutic strategies targeting extracellular pathologies like Abeta, as well as intracellular pathologies like Tau. These portals offer promising avenues for further research and development in the pursuit of effective treatments for Alzheimer's disease.

**Conclusion:**

These newly identified Portal receptors may enable unprecedented control over biodistribution, enhancing CNS delivery, cell‐type specificity, regional targeting, and reduced peripheral effects. By substantially expanding the known Portal landscape, our work provides a foundation for developing biologics with precisely engineered tissue distribution profiles that are sculpted to meet the intended product profiles for specific therapeutic approaches. The Transcytosome represents a crucial step toward realizing the full therapeutic potential of biologics in CNS diseases such as Alzheimer’s.

